# Implantation of cardiac resynchronization therapy defibrillator with left bundle branch pacing in a patient with mirror-image dextrocardia: a case report

**DOI:** 10.3389/fcvm.2026.1834563

**Published:** 2026-06-09

**Authors:** Hongwei Yi, Jia Li, Hongwei Han, Yue Bao

**Affiliations:** Department of Cardiology, Wuhan Asia Heart Hospital, Wuhan, China

**Keywords:** cardiac resynchronization therapy defibrillator, complete left bundle branch block, heart failure, left bundle branch pacing, mirror-image dextrocardia, physiological pacing

## Abstract

Mirror-image dextrocardia is a rare congenital malformation of visceral transposition, and its completely reversed cardiac anatomical structure poses great challenges to cardiac resynchronization therapy (CRT). Currently, clinical experience with left bundle branch pacing (LBBP) in such special patients remains extremely limited. This article reports a case of a 73-year-old male patient diagnosed with mirror-image dextrocardia complicated by advanced heart failure and complete left bundle branch block (CLBBB). Through preoperative mirror-image adaptation of *electrocardiogram* and digital subtraction angiography (DSA) systems, combined with reverse personalized heat shaping of the delivery sheath during the procedure, we successfully completed the implantation of an LBBP-based cardiac resynchronization therapy defibrillator (CRT-D). This case is extremely rare and provides valuable insights for the management of such patients.

## Background

Mirror-image dextrocardia is a rare congenital malformation of visceral transposition with an incidence of approximately 0.01% in the general population. Its core characteristic is that the heart is entirely located in the right thoracic cavity, with complete mirror-image reversal of the anatomical structures of the atria, ventricles, and great vessels (1). Such anatomical reversals completely subvert the spatial anatomical relationships of the normal heart, substantially increasing the difficulty and risk of cardiovascular interventional procedures. For cardiac pacing device implantation, routine operating procedures, projection positions, and target mapping methods cannot be directly applied. For patients with heart failure with reduced ejection fraction (HFrEF) complicated by CLBBB, CRT represents a Class I indication recommended by current guidelines (2), which can significantly improve cardiac function and reduce all-cause mortality. In recent years, as an emerging physiological pacing modality, LBBP achieves left ventricular electrical synchronization through direct activation of the His-Purkinje system.

It has become an important alternative to traditional biventricular pacing (BiVP), especially for patients with difficult conventional CRT implantation or non-response (3). Compared with BiVP, LBBP demonstrates potential advantages in correcting CLBBB, narrowing QRS duration, improving ventricular synchrony, and reducing lead-related complications. A 3-year follow-up study by Ge’s group confirmed that under strict electrophysiological criteria and mature implantation techniques, the long-term clinical outcomes of LBBP were significantly better than those of traditional BiVP (4). Long-term follow-up data from our center also indicate that LBBP is superior to BiVP in promoting cardiac reverse remodeling, increasing the echocardiographic super-response rate, and improving long-term prognosis (5). At present, LBBP has become a preferred physiological pacing option for CRT in our center. However, for patients with mirror-image dextrocardia, routine LBBP projection positioning, sheath shaping, target mapping, and lead fixation all face substantial challenges due to the complete reversal of anatomical structures. To date, case reports on the standardized diagnosis and treatment of this condition remain extremely scarce both domestically and internationally, with no unified standardized operative protocol available for reference. In this case, based on the unique anatomical characteristics of mirror-image dextrocardia, we completed dual mirror-image adaptation of *electrocardiogram* and imaging *prior to the procedure*. *Using reverse customized sheath shaping and mirror-image DSA*, we successfully performed LBBP-CRT-D implantation in a patient with mirror-image dextrocardia complicated by advanced heart failure. Intraoperatively, accurate capture of the left bundle branch was achieved, with ideal pacing and defibrillation parameters obtained. This case aims to summarize the diagnosis and treatment experience of such rare cases, explore the feasibility and key operative points of LBBP-CRT-D implantation, and provide new practical references for physiological resynchronization therapy in heart failure patients with complex anatomical variations.

## Case presentation

**Figure 1:**
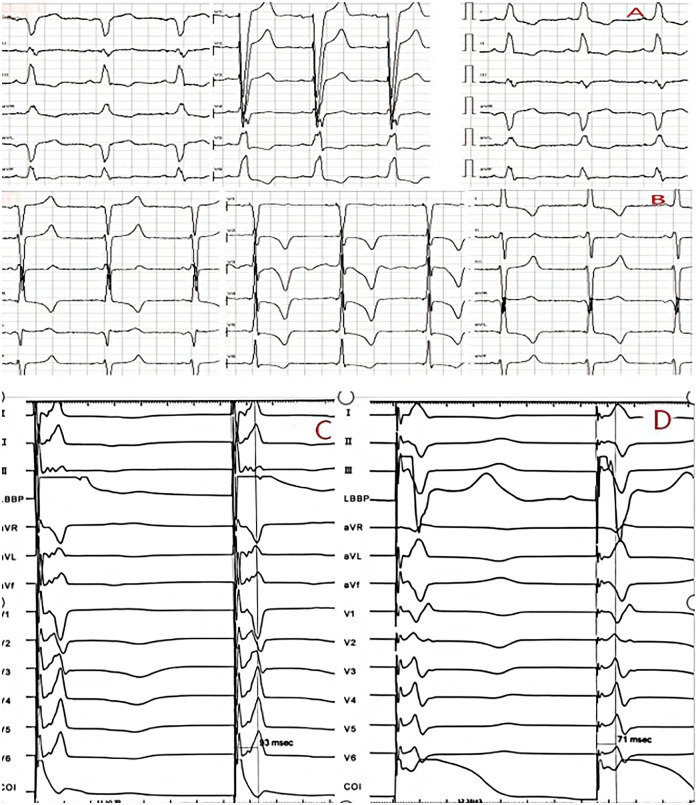
**(A)** Electrocardiograms with standard limb lead connection (left panel) and reversed limb lead connection obtained before the procedure. **(B)** Electrocardiograms with standard limb lead connection (left panel) and reversed limb lead connection obtained after the procedure. **(C,D)** Intraoperative endocardiogram: Stimulus-to-peak R-wave interval in lead V6 during His bundle pacing for correction of left bundle branch block was 93 ms **(C)**; Stimulus-to-peak R-wave interval in lead V6 during left bundle branch pacing was 71 ms **(D)**. LBBP, left bundle branch pacing; COI, current of injury.

A 73-year-old male was admitted to our hospital due to “chest tightness and shortness of breath after exertion for 1 year, aggravated for 20 days”. He had been previously healthy. Physical examination on admission revealed *crackles* at the bases of both lungs, apical impulse located in the right chest, cardiac border enlarged to the right, and bilateral lower extremity edema. Laboratory examination: N-terminal pro-B-type natriuretic peptide (NT-proBNP) was 20,587.00 pg/mL. Echocardiography: Mirror-image dextrocardia was confirmed, with left ventricular end-diastolic diameter (LVEDD) of 64 mm, left ventricular ejection fraction (LVEF) of 35%, diffuse hypokinesis of global left ventricular systolic function, no obvious valvular abnormalities, and no pericardial effusion. The e*lectrocardiogram* with reversed limb lead connections and mirror-image placement of precordial electrodes (Figure 1A): sinus rhythm, CLBBB, QRS duration 171 ms. Chest computed tomography (CT): Mirror-image dextrocardia with complete visceral transposition and small left pleural effusion. The patient had sinus rhythm, LVEF ≤35%, complicated by CLBBB with QRS duration ≥150 ms. Heart failure symptoms continued to worsen despite optimized guideline-directed medical therapy (GDMT), meeting a Class I indication for CRT-D implantation. Given the patient’s mirror-image dextrocardia, the left ventricular lead delivery route and coronary venous anatomy required for conventional BiVP were difficult to implement due to mirror-image reversal. Therefore, LBBP was directly selected as the primary resynchronization strategy, with conventional CRT as a rescue strategy if LBBP morphology was unsatisfactory. After obtaining written informed consent from the patient and his family, LBBP-CRT-D implantation was planned.

**Figure 2:**
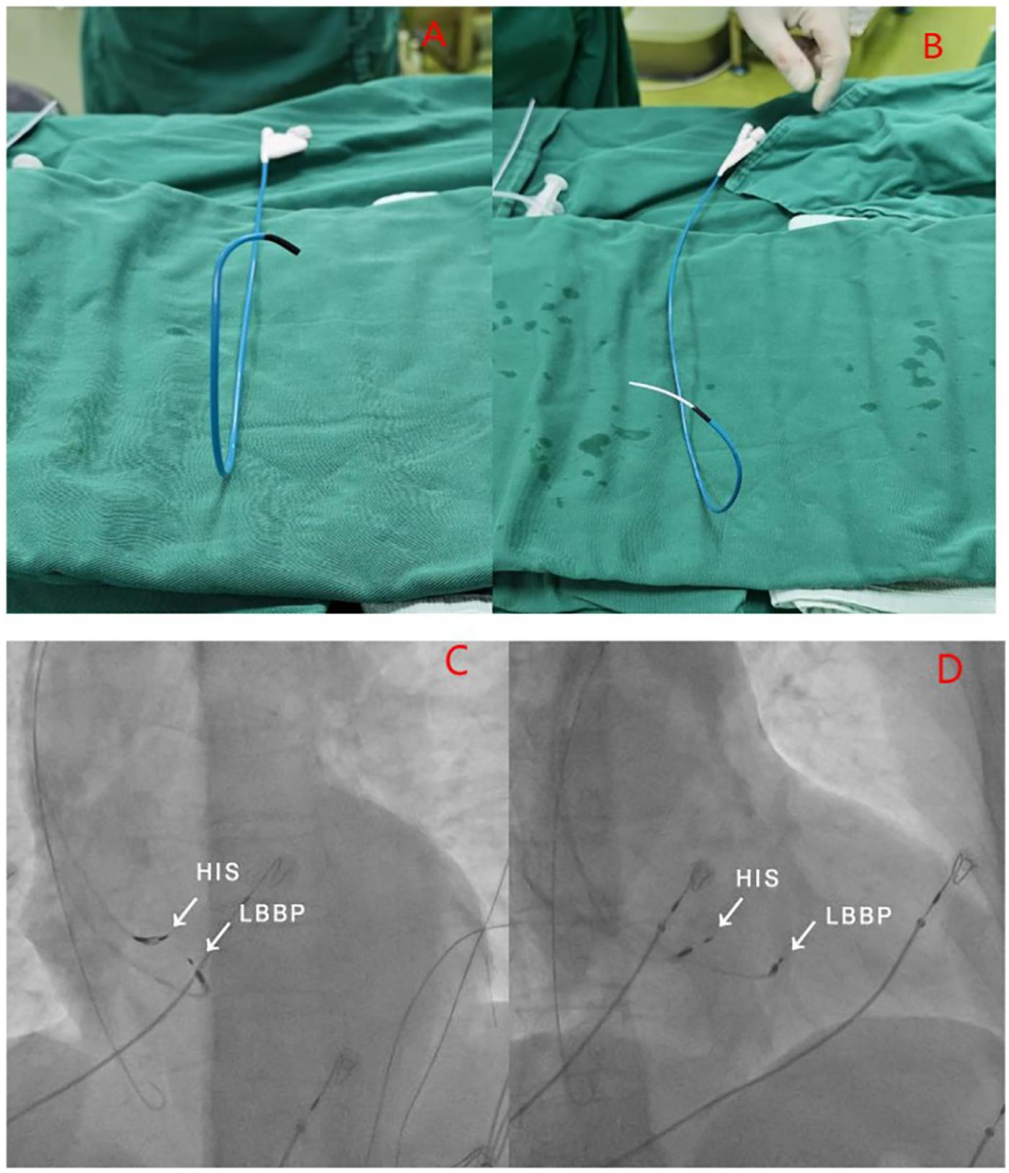
**(A,B)** Native C315HIS sheath **(A)** and reshaped sheath **(B)**. **(C,D)** Right anterior oblique 45° **(C)** and left anterior oblique 30° **(D)** views show the positions of the left bundle branch lead (labeled LBBP) and His bundle (labeled HIS).

**Figure 3:**
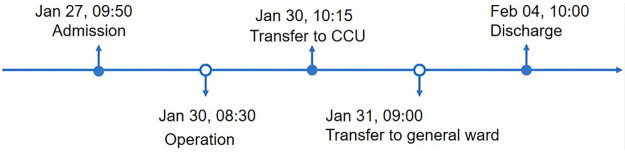
The timeline of the patient's *clinical course during hospitalization*.

Operative Procedure: *Prior to the procedure, the DSA system was preset to left-right flipped mirror-image mode. Subtraction was applied under fluoroscopy to eliminate interference from bone and soft tissue, clearly delineating cardiac chamber contours, lead course, and sheath position. Mirror-image flipping restored an operating perspective close to that of normal levocardia, significantly reducing the difficulty of anatomical positioning.* As the patient's superior vena cava coursed through the left thoracic cavity, the right axillary vein was selected as the venous access. The Seldinger technique was used to successfully puncture the right axillary vein and insert a guidewire. Under fluoroscopy, the guidewire smoothly reached the *morphological* right atrium and inferior vena cava via the superior vena cava in the left thoracic cavity. A device pocket was then created in the right pectoral region. A peel-away sheath was inserted along the guidewire, and a Medtronic 6935M defibrillation lead (Medtronic, Minneapolis, MN, USA) was delivered through the sheath to the *morphological* right ventricle, with active fixation completed at the right ventricular apex. Test parameters: pacing threshold 0.5 V, R-wave sensitivity 28 mV, impedance 450 Ω. The lead was connected to a temporary pacemaker for standby. Subsequently, a Medtronic C315HIS delivery sheath (Medtronic, Minneapolis, MN, USA) was advanced through the peel-away sheath to the *morphological* right ventricle. Contrast medium (iohexol) was injected to visualize the right ventricular cavity and the apex of the tricuspid septal leaflet. Due to the complete anatomical reversal in mirror-image dextrocardia, the native curvature of the C315HIS sheath was completely mismatched with the course of the interventricular septum. Drawing on the mature experience of intraoperative heat shaping of sheaths reported by Tetsuro Shimura (6), we used a sterile hair dryer in high-temperature, low-airflow mode to heat the sheath tip for 15–20 s, followed by manual reverse shaping. The tip curvature was adjusted to a rightward bend opposite to the native curvature (Figures 2A,B) to adapt to the course of the interventricular septum under mirror-image anatomy. The shaped C315HIS sheath and Medtronic 3830 active fixation lead (Medtronic, Minneapolis, MN, USA) were delivered to the right ventricle. The sheath was rotated clockwise and slightly retracted to make its tip closely appose the interventricular septum, and a clear His bundle potential was mapped. The CLBBB pattern was completely corrected under high-output pacing (10 V, pulse width 0.4 ms), and the interval from His bundle potential to the R-wave peak in lead V6 was measured as 89 ms. Based on the His bundle mapping position and right ventricular apex imaging as references, the shaped C315HIS sheath was rotated clockwise to appose its tip to the target area of the mid-interventricular septum. However, stable fixation of the lead at the target position was difficult after multiple rotations, which was considered related to poor atrial fulcrum of the sheath, *insufficient* axial angle between the tip and the interventricular septum, and insufficient support. Therefore, the original peel-away sheath (15 cm) was replaced with a MicroPort PLS-2507 long sheath (25 cm, MicroPort, Shanghai, China) to adjust the atrial fulcrum and axial direction of the C315HIS sheath and enhance sheath support. After two adjustments of sheath position with lead unscrewing and re-screwing for mapping, the lead was successfully fixed in the left posterior fascicular region (Figures 2C,D). The pacing electrocardiogram showed dominant negative deflections in the inferior leads and an rSR' pattern in lead V1. The interval from the pacing stimulus to the R-wave peak in lead V6 (Stimulus-to-left ventricular activation time, Stim-LVAT) was measured as 71 ms (Figures 1C,D). Test parameters: pacing threshold 0.5 V, R-wave sensitivity 19.0 mV, impedance 790 Ω. Finally, another C315HIS delivery sheath was inserted along the third guidewire. Using the same reverse shaping method, the sheath was rotated to deliver a second Medtronic 3830 active fixation lead to the *morphological* right atrium, positioned at the mid-interatrial septum with active fixation. Test parameters: threshold 0.7 V (pulse width 0.5 ms), P-wave sensitivity 3.0 mV, impedance 600 Ω. All parameters met acceptance criteria. The three leads were connected to a Medtronic DTMA2D4 CRT-D pulse generator (Medtronic, Minneapolis, MN, USA). The pulse generator was implanted in the right anterior chest pocket, and the wound was closed in layers. Total operative time was 110 min, with fluoroscopy time of 28 min. The patient remained hemodynamically stable throughout the procedure, with no procedure-related complications such as pneumothorax, pericardial effusion, vascular injury, or myocardial perforation. The patient returned to the ward safely after surgery. Postoperative electrocardiogram showed LBBP pacing rhythm with QRS duration shortened to 125 ms (Figure 1B). Follow-up *echocardiography* at 1 month demonstrated LVEF increased to 51% and LVEDD reduced to 55 mm. The timeline of the patient's management during hospitalization is summarized in Figure 3.

## Discussion

Mirror-image dextrocardia is a rare congenital malformation of visceral transposition with the core anatomical feature of complete mirror-image reversal of cardiac and great vessel structures, often accompanied by visceral transposition; some patients may have concurrent congenital cardiovascular malformations. This special anatomical variation poses substantial challenges to cardiac resynchronization therapy defibrillator implantation. For patients with HFrEF complicated by CLBBB, CRT represents the first-line treatment recommended by guidelines. Traditional BiVP has certain limitations in clinical practice due to constraints of coronary venous anatomy, left ventricular lead complications, and non-physiological activation sequence. As an important form of physiological conduction system pacing, LBBP can directly activate the left bundle branch to achieve a more physiological ventricular activation sequence. It exerts favorable effects in improving ventricular synchrony, narrowing QRS duration, and promoting left ventricular reverse remodeling, and has become an important alternative in the field of CRT (6). However, no completed large-scale long-term randomized controlled trial has confirmed that LBBP is superior to conventional CRT, and relevant guidelines do not recommend LBBP as the first choice to replace BiVP. Therefore, both techniques are currently clinically available resynchronization strategies and should be selected individually according to the patient’s anatomical characteristics, procedural conditions, and operator experience. Currently, relevant cases of LBBP-CRT-D in patients with mirror-image dextrocardia remain extremely scarce, mainly restricted by three major technical difficulties: first, the DSA projection field of view is opposite to routine operative habits, which is prone to cause anatomical positioning deviation; second, the curvature of conventional LBBP sheaths (such as C315HIS) is designed only for levocardia and cannot match the course of the interventricular septum in mirror-image dextrocardia, making effective support difficult; third, target mapping and lead fixation operations are technically demanding.

In this case, an individualized adaptation protocol was formulated to address the above difficulties, and LBBP-CRT-D implantation was successfully completed. The main technical highlights are as follows: First, reversed limb lead connections and mirror-image placement of precordial electrodes were adopted *prior to the procedure* to ensure accurate interpretation of pacing patterns; left-right mirror-image flipping of the DSA system was performed to restore the operative perspective of levocardia, reducing spatial positioning difficulty and operative errors while decreasing fluoroscopy time. Second, in response to the sheath anatomical mismatch, innovative heating reverse shaping was adopted to match the sheath tip curvature with the course of the mirror-image interventricular septum; a 25-cm long sheath was used in combination to optimize the atrial fulcrum and axial direction, enhance sheath support, and solve the problem of insufficient lead stability. Third, a strategy of “His bundle mapping+right ventricular angiography” was adopted. The target area was initially located according to the line connecting the His bundle and right ventricular apex, and the Stim-LVAT interval was used as the criterion to achieve accurate capture of the left bundle branch and obtain ideal pacing parameters. Short-term postoperative follow-up showed significant improvement in the patient's cardiac function, confirming the safety and effectiveness of this surgical approach.

Compared with existing literature, this study has unique clinical value: it represents the first detailed report on the application of LBBP-CRT-D in patients with mirror-image dextrocardia, enriching the evidence for diagnosis and treatment of rare cases; an integrated protocol of “dual mirror-image adaptation+sheath reverse shaping+long sheath support” is proposed, which has strong reproducibility and provides practical reference for similar cases; simultaneously, it achieves heart failure resynchronization therapy and primary prevention of sudden cardiac death, in accordance with guideline recommendations, with prominent clinical practical significance. This study has certain limitations: as a single-center case report, the sample size is limited; only short-term follow-up at 1 month post-surgery was completed, with lack of data on long-term cardiac function, pacing parameter stability, and long-term complications. Long-term efficacy and lead stability require further prolonged follow-up. In addition, head-to-head comparison between LBBP and conventional CRT still relies on large-scale randomized controlled trials, and the present conclusions cannot be directly generalized to the general levocardia population. In summary, LBBP-CRT-D implantation in patients with mirror-image dextrocardia complicated by advanced heart failure and CLBBB is technically demanding and requires profound anatomical knowledge and proficient operative skills from the interventional cardiologist. With preoperative mirror-image adaptation, intraoperative reverse sheath shaping, and mirror-image fluoroscopy, the procedure can be performed safely and effectively. This case provides a standardized and feasible LBBP-CRT-D implantation strategy for heart failure patients with complex anatomical variations such as mirror-image dextrocardia, and offers a valuable reference for other complex cardiovascular interventions.

## Data Availability

The original contributions presented in the study are included in the article/Supplementary Material, further inquiries can be directed to the corresponding authors.
